# Cancer incidence and mortality in Chukotka, 1997–2010

**DOI:** 10.3402/ijch.v72i0.20470

**Published:** 2013-03-19

**Authors:** Alexey A. Dudarev, Valery S. Chupakhin, Jon Øyvind Odland

**Affiliations:** 1 Hygiene Department, Northwest Public Health Research Centre, St. Petersburg, Russia; 2 AMAP Secretariat, Tromso University, Tromso, Norway

**Keywords:** Chukotka, Russian Arctic, general population, cancer incidence, epidemiology

## Abstract

**Objectives:**

The general aim was to assess cancer incidence and mortality among the general population of Chukotka in 1997–2010 and to compare it with the population of Russia.

**Methods:**

Cancer data were abstracted from the annual statistical reports of the P.A. Hertzen Research Institute of Oncology in Moscow. The annual number and percent of cases, crude and age-standardized cancer incidence (ASIR) and mortality (ASMR) rates per 100,000 among men and women in the Chukotka Autonomous Okrug were determined for the period 1997–2010 for incidence and 1999–2010 for mortality. Two years’ data were aggregated to generate temporal trends during the period. In age-standardization, the Segi-Doll world standard population used by the International Agency for Research on Cancer was used.

**Results:**

The higher incidence and mortality rate of cancer (all sites combined) among men compared to women, which was observed in Russia nationally, was reflected also in Chukotka, although the difference between men and women was not statistically significant. Overall, the patterns of cancer sites are similar between Chukotka and Russia, with cancer of the lung/trachea/bronchus and stomach occupying the top ranks among men. Oesophageal cancer is common in Chukotka but not in Russia, whereas prostate cancer is common in Russia but not in Chukotka. Among women, breast cancer is either the commonest or second commonest cancer in terms of incidence or mortality in both Chukotka and Russia. Cancer of the lung/trachea/bronchi ranks higher in Chukotka than in Russia. The rate of cancer incidence and mortality for all sites combined during the 13-year period was relatively stable in Russia. Dividing the period into two halves, an increase among both men and women was observed in Chukotka for all sites combined, and also for colorectal cancer.

**Conclusions:**

This paper presents previously unavailable cancer epidemiological data on Chukotka. They provide a basis for comparative studies across circumpolar regions and countries. With its small population, cancer rates in Chukotka tend to be highly unstable and fluctuate widely from year to year. Even when aggregated over a decade or more, only broad conclusions regarding patterns and trends can be made regarding some of the commonest cancer sites, or with all sites combined. Chukotka experienced substantial social and economic dislocations during the period under study, which could conceivably affect risk factor distribution and the quality of medical care.

Little information on the epidemiology of cancer in the Russian Arctic is available in English. This study is the first assessment of cancer incidence in the general population of Chukotka during the last decade.

## Materials and methods

Information was collected from the official statistical collections of the P.A. Hertzen Research Institute of Oncology in Moscow, which annually publishes a report entitled “Malignant neoplasms in Russia (incidence and mortality)” (1–11), covering the whole of Russia and its regions, based on reports in the regional oncology centres.

The number and percent of cases by age, sex and site were extracted from the reports, covering the 1997–2010 period for incidence and 1999–2010 period for mortality. Sex-specific crude and age-standardized cancer incidence (ASIR) and mortality (ASMR) rates and their 95% confidence interval (95% CI) for the Chukotka Autonomous Okrug and the Russian Federation were computed, using the Segi-Doll world standard population (12) used by the International Agency for Research on Cancer. To establish temporal trends over this period, the number of cases over 2-year intervals was averaged.

## Results

Appendix Tables (I–IV) present the average number of cases, crude and age-standardized rates of cancer by site in Chukotka and the whole of Russia, among men and women separately. For all sites combined, the ASIR among Chukotka men was 277 per 100,000 (95% CI: 185, 378), compared to 211 per 100,000 (95% CI: 150, 288) among women, which, statistically, is not significantly different. The ASIR among Russian men (269 per 100,000, 95% CI: 267.5, 269.8) was significantly higher than among Russian women (193 per 100,000, 95% CI: 192.9, 194.6).

The ASMR among Chukotka men was 200 per 100,000 (95% CI: 114, 286), compared to 129 per 100,000 (95% CI: 70, 189) among women. In the whole of Russia, the ASMR among men was 188 per 100,000 (95% CI: 187.3, 189.2), considerably higher than among women, with a rate of 93 per 100,000 (95% CI: 92.9, 94.0).

For both incidence and mortality, the differences between Chukotka and Russia are not statistically significant, taking into consideration the small number of cases in Chukotka and the wide confidence intervals associated with its rates.

The top 5 ranking cancers in terms of incidence and mortality among men and women in either Chukotka or Russia are shown in Table I.

**Table I:** Top 5 ranking cancers in Chukotka and Russia, 1997–2010

Incidence Site	Chukotka		Russia		Mortality Site	Chukotka		Russia	
			
	Percent	Rank	Percent	Rank		Percent	Rank	Percent	Rank
	Male					Male			
Lung/trachea/bronchi	26.3	1	22.4	1	Lung/trachea/bronchi	32.0	1	28.9	1
Stomach	12.1	2	11.4	2	Stomach	13.3	2	14.3	2
Lymphoid/HT	6.3	3	4.8	5	Oesophagus	7.3	3	3.3	10
Oesophagus	5.8	4	2.6	11	Liver/bile ducts	6.1	4	3.0	13
Rectum/RS/anus	5.4	5	4.6	6	Rectum/RS/anus	5.3	5	5.1	5
Colon	4.5	7	5.3	4	Colon	5.0	6	5.3	3
Prostate	2.7	9	7.5	3	Prostate	1.4	15	5.3	4
	Female					Female			
Breast	22.7	1	19.7	1	Lung/trachea/bronchi	21.1	1	6.5	4
Lung/trachea/bronchi	9.1	2	4.1	8	Breast	11.6	2	17.0	1
Cervix	9.1	3	5.2	5	Stomach	11.2	3	12.7	2
Colon	7.7	4	6.9	3	Colon	10.4	4	8.9	3
Rectum/RS/anus	5.9	5	4.8	7	Oesophagus	5.5	5	1.2	17
Stomach	5.6	6	7.8	2	Rectum/RS/anus	4.0	8	6.4	5
Uterus	5.0	7	6.8	4	Uterus	4.4	6	4.7	9

Among men, cancer of the lung/trachea/bronchi and cancer of the stomach, whether in terms of incidence or mortality, are predominating in both Chukotka and Russia. Eosphageal cancer is common in Chukotka but not in Russia, whereas prostate cancer is common in Russia but not in Chukotka.

Among women, breast cancer is either the commonest or second commonest cancer in terms of incidence or mortality in both Chukotka and Russia. Cancer of the lung/trachea/bronchi ranks higher in Chukotka than in Russia.

**Fig. 1:**
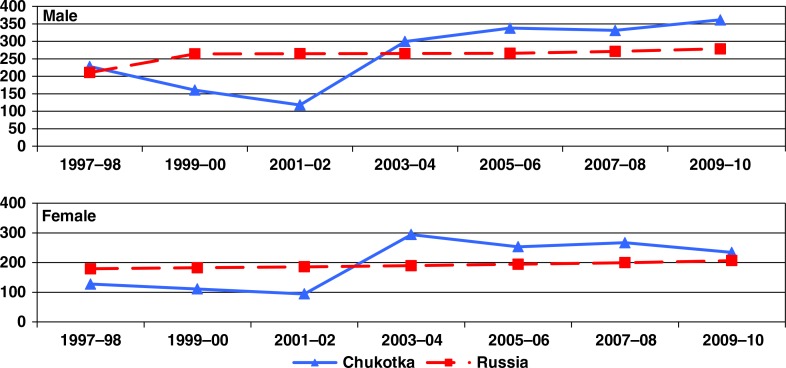
Age-standardized incidence rates (ASIR per 100,000) of cancer (all sites combined) among men and women in Chukotka and Russian Federation, 1997–2010.

The rate of cancer incidence for all sites combined during the 13-year period was relatively stable in Russia (Fig. 1), while in Chukotka it increased by 80% among men between the 1997–2002 and 2003–2010 periods, and by 150% among women between the two periods.

**Fig. 2:**
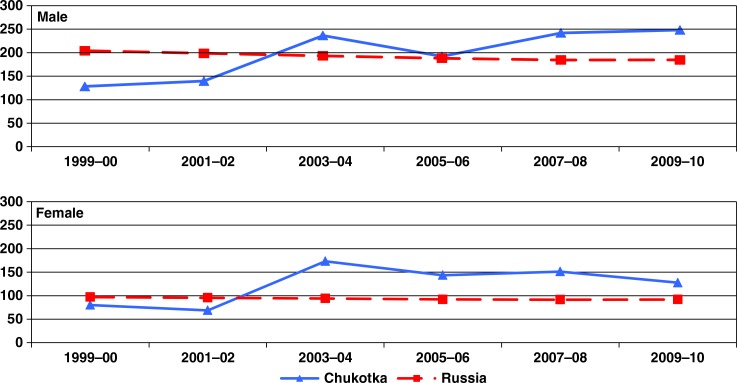
Age-standardized mortality rates (ASMR per 100,000) of cancer (all sites combined) among men and women in Chukotka and Russian Federation, 1999–2010.

For mortality, the rate for all sites combined also remained stable in Russia, but showed an 85% increase among men and a doubling among women comparing the 1999–2002 and 2003–2010 periods (Fig. 2).

In terms of individual cancer sites, no single site stands out as having changed significantly during the study period in Russia. In Chukotka, there has been a substantial increase among both men and women in the rates of some cancers between the two periods: colon among women (by 150%) and rectum/recto-sigmoid/anus among men (by 170%).

## Discussion

This paper presents epidemiological data on cancer in one of Russia's most remote Arctic regions, which have not been previously available in English publications outside Russia. They provide a basis for comparative studies across circumpolar regions and countries.

Cancer mortality is often used as a proxy for incidence if the latter is not available. Both are available for Chukotka and Russia. Mortality is a function of both incidence and survival, which depends on the access to and quality of health care.

With its small population, cancer rates in Chukotka tend to be highly unstable and fluctuate widely from year to year. Even when aggregated over a decade or more years, only broad conclusions regarding patterns and trends can be made regarding some of the commonest cancer sites, or with all sites combined.

Chukotka experienced substantial social and economic dislocations during the period under study (see Dudarev et al. in this Special Issue). Such changes could conceivably affect risk factor distribution and the quality of medical care – which in turn affects not only survival, but also detection – and thus the cancer morality and incidence rates. There is also a major re-distribution of the population, substantially increasing the proportion of indigenous people within the total regional population due to out-migration of non-indigenous people. The epidemiology of cancer among Chukotka natives is the subject of a companion paper in this Special Issue.

## Appendix

**Appendix Table I:** Average annual number of cases, percent, crude and age-standardized cancer incidence rates (ASIR) per 100,000 among men in Chukotka and Russian Federation, 1997–2010

	Chukotka					Russia				
		
Male	Cases/year	%	Crude	ASIR	95% CI	Cases/year	%	Crude	ASIR	95% CI
Lips	0.5	0.8	1.4	1.5	0–4.3	3242.5	1.5	4.8	3.9	3.6–3.8
Tongue	0.9	1.4	2.9	2.7	0–7.2	1717.2	0.8	2.6	2.1	2–2.2
Salivary glands	0.3	0.4	0.9	0.6	0–1.8	575	0.3	0.9	0.7	0.6–0.8
Other parts of oral cavity	1	1.6	3.7	6.9	0–19.7	2637.1	1.2	4	3.2	3.1–3.3
Oropharynx	0.8	1.2	2.4	4.6	0–13.9	1681.8	0.8	2.5	2.1	1.9–2.1
Nasopharynx	0.4	0.7	1.5	1.5	0–4.3	372.5	0.2	0.6	0.5	0.4–0.5
Laryngopharynx	0.7	1	2.3	4.5	0–13	1524.6	0.7	2.3	1.9	1.8–2
Oesophagus	3.6	5.8	11.9	13.2	0–28.3	5648.4	2.6	8.5	6.9	6.6–7
Stomach	7.8	12.1	25.5	47.5	2.3–88.3	25261.1	11.4	37.8	30.3	29.2–30
Colon	2.8	4.5	9.5	9	0–18.1	11836.6	5.3	17.8	14.2	14.1–14.6
Rectum/RSJ/anus	3.3	5.4	11.8	16.1	0–37.4	10283.5	4.6	16.6	13.2	13.1–13.6
Liver/bile ducts	2.1	3.2	7.3	10.1	0–24.6	3800.1	1.7	5.7	4.6	4.4–4.7
Pancreas	1.5	2.3	5.4	7.9	0–21.5	6939.8	3.1	10.4	8.4	8.1–8.5
Larynx	1.5	2.6	4.7	4	0–10	6555.6	3	9.8	7.9	7.6–8
Lung/trachea/bronchi	16.7	26.3	56.2	63.8	24.1–106.9	49480.5	22.4	74.2	59.6	58–59.1
Bones/cartilages	0.6	0.9	2	4.6	0–14	1170.5	0.5	1.8	1.6	1.4–1.6
Skin melanoma	0.5	0.8	1.9	1.2	0–3.8	2664.5	1.2	4	3.2	3.1–3.4
Other MSN	1.7	2.6	6	10.2	0–24.8	20505.7	9.2	30.8	24.7	24.5–25.2
Connective/other ST	0.4	0.7	1.2	1.2	0–2.6	1528.1	0.7	2.3	2	1.9–2.1
Prostate	1.8	2.7	6.3	10.1	0–24.6	16839	7.5	25.4	20.1	20.5–21.1
Kidneys	3	4.6	11.2	12.4	0–28.9	8654.1	3.9	13	10.5	10.5–11
Urinary bladder	1.8	2.6	6.5	9.5	0–24.4	9911.3	4.5	14.9	11.9	11.7–12.2
Brain/CNS	1.1	1.7	3.9	3.4	0–8.9	3015.3	1.4	4.6	4	4–4.3
Thyroid gland	0.8	1.1	2.9	2.2	0–6.2	1191	0.5	1.8	1.4	1.4–1.6
Lymphoid/HT	3.8	6.3	11.8	10.3	0–21.8	10690.1	4.8	16.1	14.1	13.9–14.4
Other sites	4.1	6.5	0	0	0	14013.8	6.3	0	0	0
All sites	63.3	100	214.5	277.3	185.2–378.4	221739.3	100	332.8	269	267.5–269.8

RSJ=rectum-sigmoid junction, MSN=malignant skin neoplasm, ST=soft tissues, HT=hematopoietic tissues, CNS=central nervous system.

**Appendix Table II:** Average annual number of cases, percent, crude and age-standardized cancer incidence rates (ASIR) per 100,000 among women in Chukotka and Russian Federation, 1997–2010

	Chukotka					Russia				
		
Female	Cases/year	%	Crude	ASIR	95% CI	Cases/year	%	Crude	ASIR	95% CI
Lips	0.0	0.0	0.0	0.0	0–0	1063.0	0.4	1.4	0.6	0.6–0.6
Tongue	0.2	0.3	0.6	0.4	0–1.3	535.0	0.2	0.7	0.4	0.4–0.5
Salivary glands	0.1	0.1	0.3	1.1	0–3.6	524.0	0.2	0.7	0.4	0.4–0.5
Other parts of oral cavity	0.4	0.8	1.5	2.0	0–5.9	677.4	0.3	0.9	0.5	0.5–0.6
Oropharynx	0.3	0.4	0.9	0.6	0–1.3	195.2	0.1	0.3	0.2	0.2–0.2
Laryngopharynx	0.0	0.0	0.0	0.0	0–0	107.2	0.0	0.1	0.1	0.1–0.1
Oesophagus	1.5	2.9	5.4	9.3	0–25.2	1747.0	0.7	2.3	1.1	1–1.1
Stomach	2.9	5.6	10.7	12.3	0–26.1	19100.5	7.8	24.9	13.0	12.5–12.9
Colon	4.3	7.7	16.0	18.8	0–40.2	17046.5	6.9	22.3	11.7	11.6–12
Rectum/RSJ/anus	3.1	5.9	11.3	13.0	0–28.7	11803.3	4.8	15.4	8.4	8.3–8.6
Liver/bile ducts	0.6	1.1	1.9	3.4	0–9.3	2986.3	1.2	3.9	2.1	2–2.1
Pancreas	0.6	1.0	2.4	2.6	0–7.1	6644.2	2.7	8.7	4.4	4–4.2
Larynx	0.5	0.8	2.1	2.2	0–6.6	390.2	0.2	0.5	0.3	0.3–0.3
Lung/trachea/bronchi	4.9	9.1	18.6	24.9	1.9–51	10079.8	4.1	13.1	7.0	6.8–7.1
Bones/cartilages	0.3	0.4	0.9	1.2	0–2.5	923.7	0.4	1.2	1.0	0.9–1
Skin melanoma	1.1	2.1	3.6	2.3	0–5.5	4427.8	1.8	5.8	3.7	3.7–3.9
Other MSN	2.5	4.4	9.3	9.4	0–20	33018.8	13.3	43.1	21.8	21.7–22.2
Connective/other ST	0.3	0.6	1.4	1.4	0–4.5	1690.8	0.7	2.2	1.6	1.5–1.7
Breast	12.3	22.7	45.4	40.1	15–67.7	48852.3	19.7	63.8	40.6	40.7–41.5
Cervix uteri	4.9	9.1	18.3	17.4	0.1–36.4	12999.8	5.2	17.0	12.0	11.9–12.3
Corpus uteri	2.8	5.0	10.2	8.6	0–19.6	16964.5	6.8	22.1	13.9	13.8–14.3
Ovaries	2.1	3.9	7.4	9.2	0–23.5	12192.3	4.9	15.9	10.5	10.3–10.7
Kidneys	1.3	2.3	5.3	4.6	0–11.9	6818.3	2.7	8.9	5.5	5.5–5.8
Urinary bladder	0.5	0.9	2.0	2.2	0–6.9	2621.6	1.1	3.4	1.7	1.7–1.8
Brain/CNS	0.3	0.6	1.4	1.3	0–4.1	2841.3	1.1	3.7	3.1	3–3.2
Thyroid gland	2.2	3.9	8.2	5.7	0–13.3	6990.9	2.8	9.1	6.6	6.6–7
Lymphoid/HT	2.3	4.2	8.3	10.1	0–24.1	10997.6	4.4	14.4	10.4	10.2–10.7
Other sites	2.3	4.1	0.0	0.0	0–0	13608.8	5.5	0.0	0.0	0.0
All sites	54.5	100.0	201.8	211.8	150.4–288.5	248091.9	100.0	323.7	192.5	192.9–194.6

RSJ=rectum-sigmoid junction, MSN=malignant skin neoplasm, ST=soft tissues, HT=hematopoietic tissues, CNS=central nervous system.

**Appendix Table III:** Average annual number of deaths, percent, crude and age-standardized cancer mortality rates (ASMR) per 100,000 among men in Chukotka and Russian Federation, 1999–2010

	Chukotka					Russia				
		
Male	Deaths/year	%	Crude	ASMR	95% CI	Deaths/year	%	Crude	ASMR	95% CI
Lips/oral cavity/pharynx	1.8	4.4	6.4	6.9	0–15.5	7103.9	4.5	10.7	8.6	8.4–8.8
Oesophagus	3.1	7.3	10.2	13.2	0–29.8	5247.5	3.3	7.9	6.3	6.2–6.5
Stomach	5.4	13.3	18.7	41.3	0–85.3	22389.5	14.3	33.7	26.6	26.2–27
Small intestine	0.1	0.2	0.3	0.2	0–0.6	519.3	0.3	0.8	0.6	0.6–0.7
Colon	2	5	7.1	11.2	0–28.6	8255.9	5.3	12.4	9.8	9.6–10
Rectum/RSJ/anus	2.2	5.3	7.7	9.7	0–24.1	8014	5.1	12.1	9.5	9.3–9.7
Liver/bile ducts	2.5	6.1	8	11.3	0–27	4636.5	3	7	5.6	5.4–5.7
Pancreas	1.5	3.6	5.1	8.2	0–21.5	7158.5	4.6	10.8	8.6	8.3–8.8
Other digestive organs	0.2	0.5	0.6	0.3	0–1	1536.3	1	2.3	1.8	1.7–1.9
Larynx	1.5	3.6	4.9	6.4	0–15.2	4914.5	3.1	7.4	5.9	5.7–6.1
Lung/trachea/bronchi	13.1	32	45.3	61.4	17.9–97.8	45412.6	28.9	68.3	54.2	53.7–54.7
Other respiratory organs	0.3	0.7	0.9	0.7	0–2	1064.7	0.7	1.6	1.3	1.2–1.4
Bones/cartilages	0.3	0.7	0.7	1.1	0–2.9	1121.5	0.7	1.7	1.4	1.3–1.5
Skin melanoma	0.5	1.4	2	1.3	0–3.9	1335.5	0.9	2	1.6	1.5–1.7
Other MSN	0	0	0	0	0–0	757.7	0.5	1.2	0.9	0.8–1
Connective/other ST	0.6	1.6	2.1	1.7	0–4.9	1451.3	0.9	2.2	1.8	1.7–1.9
Prostate	0.5	1.4	2.1	2.6	0–7	8248.6	5.3	12.4	9.8	9.6–10
Other male genital organs	0.4	0.9	1.4	1.3	0–3	710.9	0.5	1.1	0.9	0.8–0.9
Kidneys	1.1	2.7	4	5	0–12.9	4841.1	3.1	7.3	5.8	5.7–6
Urinary bladder	1	2.4	3.5	3.7	0–7.7	5628.7	3.6	8.5	6.7	6.5–6.9
Other urinary organs	0	0	0	0	0–0	355.5	0.2	0.5	0.5	0.4–0.5
Brain/CNS	0.9	2.2	3.2	2.4	0–5.7	3098.8	2	4.7	4	3.8–4.1
Lymphoid/HT	1.4	3.2	4.6	4.6	0–11.5	7266.6	4.6	10.9	9.1	8.9–9.4
Other sites	0.7	1.8	0	0	0	5828.6	3.7	0	0	0
All sites	40.9	100	141.1	199.8	113.5–286.1	15689.8	100	236	188.3	187.3–189.2

RSJ=rectum-sigmoid junction, MSN=malignant skin neoplasm, ST=soft tissues, HT=hematopoietic tissues, CNS=central nervous system.

**Appendix Table IV:** Average annual number of deaths, percent, crude and age-standardized cancer mortality rates (ASMR) per 100,000 among women in Chukotka and Russian Federation, 1999–2010

	Chukotka					Russia				
		
Female	Deaths/year	%	Crude	ASMR	95% CI	Deaths/year	%	Crude	ASMR	95% CI
Lips/oral cavity/pharynx	0.3	1.1	0.9	0.7	0–2	1563.2	1.2	2	1.1	1.1–1.2
Oesophagus	1.4	5.5	5	7.8	0–19.6	1569.3	1.2	2	0.9	0.9–1
Stomach	2.9	11.2	10.7	13.4	0–30.2	16772.4	12.7	21.9	10.9	10.7–11.1
Small intestine	0.1	0.4	0.4	1	0–3	612.2	0.5	0.8	0.4	0.4–0.4
Colon	2.6	10.4	9.8	14.7	0–31.9	11745.1	8.9	15.4	7.4	7.2–7.5
Rectum/RSJ/anus	1	4	3.6	4.1	0–11	8422.8	6.4	11	5.4	5.3–5.6
Liver/bile ducts	0.9	3.7	3.4	4	0–9.9	3613.7	2.7	4.7	2.4	2.3–2.5
Pancreas	0.6	2.6	2.6	3.3	0–8.5	6834	5.2	8.9	4.4	4.3–4.5
Other digestive organs	0.3	1	1	1.6	0–4.7	2691.8	2	3.5	1.7	1.6–1.8
Larynx	0.3	1	1.1	0.6	0–1.9	239.5	0.2	0.3	0.2	0.2–0.2
Lung/trachea/bronchi	5.5	21.1	21	28.8	2.9–49.1	8555.2	6.5	11.2	5.7	5.6–5.8
Other respiratory organs	0.1	0.3	0.4	0.7	0–2.2	597.3	0.5	0.8	0.5	0.4–0.5
Bones/cartilages	0.1	0.5	0.4	0.6	0–1.9	819.2	0.6	1.1	0.7	0.6–0.8
Skin melanoma	0.5	1.6	1.7	1	0–2.9	1616.1	1.2	2.1	1.2	1.2–1.3
Other MSN	0.1	0.3	0.4	1.2	0–3.6	930.8	0.7	1.2	0.6	0.5–0.6
Connective/other ST	0.3	1.2	1.1	1.4	0–4.1	1605.9	1.2	2.1	1.3	1.2–1.4
Breast	3	11.6	11.6	12.6	0–27.5	22467.4	17	29.3	17.2	17–17.4
Cervix uteri	1.1	4.3	4.3	5.7	0–14.7	6156.8	4.7	8	5.1	4.9–5.2
Corpus uteri	1.2	4.4	4.5	5.1	0–12.3	6236.4	4.7	8.1	4.4	4.3–4.5
Ovaries	0.7	3	2.7	6.3	0–18	7429.2	5.6	9.7	5.7	5.6–5.9
Kidneys	0.4	1.2	1.4	1.9	0–5.1	3035	2.3	4	2.1	2–2.2
Urinary bladder	0.4	1.5	1.4	2.2	0–6.5	1390.6	1.1	1.8	0.8	0.7–0.8
Other urinary organs	0	0	0	0	0	195.3	0.1	0.2	0.1	0.1–0.2
Brain/CNS	0.5	2	2.3	1.6	0–4.7	2885.1	2.2	3.8	2.8	2.7–2.9
Lymphoid/HT	0.7	2.8	2.8	4.4	0–11.2	7087.3	5.4	9.2	5.8	5.7–6
Other sites	0.7	3.3	0	0	0	7097.4	5.4	0	0	0
All sites	25.6	100	97	129	69.6–188.5	132168.8	100	172.6	93.5	92.9–94

RSJ=rectum-sigmoid junction, MSN=malignant skin neoplasm, ST=soft tissues, HT=hematopoietic tissues, CNS=central nervous system.
